# Delayed enhancement cardiac magnetic resonance imaging predicts future arrhythmic events in primary prevention ICD candidates irrespective of ischemic or non-ischemic etiology

**DOI:** 10.1186/1532-429X-13-S1-O103

**Published:** 2011-02-02

**Authors:** Peng Gao, Raymond Yee, Ellie Hogg, John Stirrat, David Scholl, Lorne Gula, Andrew D Krahn, Allan Skanes, Peter Leong-Sit, George Klein, James A White

**Affiliations:** 1 London Health Sciences Centre – Division of Cardiology, London, ON, Canada; 2 Robarts Research Institute, London, ON, Canada; 3 London Health Sciences Centre – Division of Cardiology; Robarts Research Institute; Lawson Health Research Institute, London, ON, Canada

## Introduction

Risk prediction of first sudden cardiac arrest (SCA) in patients with ischemic cardiomyopathy (ICM) and non-ischemic cardiomyopathy (NICM) is imperfect and based solely upon ejection fraction. Delayed enhancement (DE) MRI has been shown in both populations to identify those at higher risk of cardiovascular events. However, how this technique may predict arrhythmic events in ICD candidates is poorly understood, especially in those with NICM.

## Purpose

To explore the utility of DE-MRI for the prediction of arrhythmic events in candidates for primary prevention implantable cardiac defibrillators (ICD)

## Methods

125 consecutive patients with ICM (n=61) or NICM (n=64) being screened for ICD eligibility underwent DE MRI and were prospectively followed for the primary combined endpoint of appropriate ICD therapy or SCA. A composite secondary endpoint of major adverse events (SCA, appropriate ICD therapy and all-cause mortality) was also evaluated. DE-MRI was performed using a standard protocol 10 minutes following gadolinium contrast administration (Gadovist®, Bayer, Inc) on a 3T Siemens scanner. Serial short axis images were analyzed using commercial software (CMR42, Circle Cardiovascular Inc, Calgary) to determine signal volume at ≥2, 3 and 5SD thresholds above normal reference myocardium. Total scar was defined as signal ≥2SD while the difference between 3 and 5SD thresholds was defined as “borderzone”.

## Results

The mean age and ejection fraction was 61.4±11.2 yo and 26.5±7.5%, respectively. 76 patients received an ICD eventually. During a median follow-up of 412±191days, 16 (13%) of patients had a primary endpoint (15 ICD therapies and 1 aborted SCA). An additional 6 patients had death by any cause contributing to the secondary endpoint. The mean total scar mass was significantly higher in patients with both a primary outcome (65.1±27.8 g vs. 31.3±18.7 g, p<0.001) or secondary outcome (54.5±29.6 g vs. 31.6±19.1 g, p<0.001). Similarly, the respective borderzone mass was higher in both patient groups (25.3±10.8g vs. 18.3±9.8g, p=0.009) and secondary events (23.7±10.5 g vs. 18.2±9.9g p=0.02). ROC analysis revealed that total scar mass had the greatest AUC values of 0.85 (0.73~0.96, p<0.001) and 0.74 (0.63~0.86, P<0.001) for primary and secondary events respectively. Only total scar mass remained predictive of the primary or secondary outcome in multivariate analysis. In subgroup analysis of ICM and NICM patients, total scar mass was still the sole index of adverse events.

## Conclusions

Total scar mass predicts arrhythmic events in patients being considered for primary prevention ICD irrespective of an ischemic or non-ischemic etiology.

**Figure 1:**
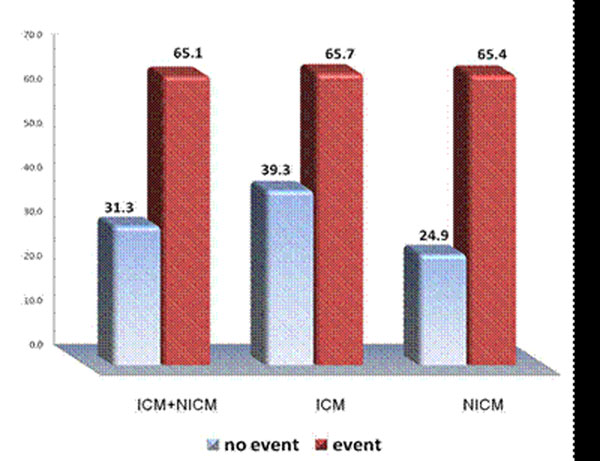
Graph showing significantly higher amount of scar mass (3SD definition) with primary events than no events in all patients, ischemic cardiomyopathy and non-ischemic cardiomyopathy group (p<0.05 respectively).

**Figure 2:**
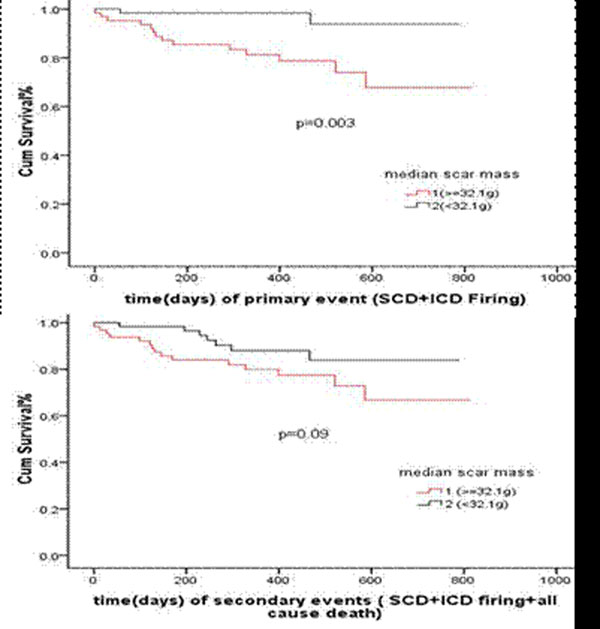
Kaplan-Meier curve for primary and secondary events. The patients were grouped by a median scar mass of 32.1g.

